# Analysis on the relationship between effort-reward imbalance and job satisfaction among family doctors in China: a cross-sectional study

**DOI:** 10.1186/s12913-022-08377-5

**Published:** 2022-08-04

**Authors:** Jinhua Chen, Yijun Wang, Wen Du, Shuyi Liu, Zhu Xiao, Yuelei Wu

**Affiliations:** Department of General Practice, Chengdu First People’s Hospital, Chengdu, 610041 China

**Keywords:** Family doctor, Primary Health Care Centres, Community, Effort-Reward imbalance, Job Satisfaction

## Abstract

**Background:**

Family doctor contract services was launched in Sichuan province in 2016. The focus was mainly on developing primary health care services but paying less attention to the work stress and job satisfaction of in-service family doctors.

**Objective:**

This study aims to explore the influencing factors of job satisfaction, and the relation between work stress indicators and job satisfaction among family physicians.

**Methods:**

An analytical online cross-sectional survey was performed among 1,105 family doctors from 23 districts and counties in Chengdu. Self-administered questionnaire was completed. Sociodemographic factors, work stress measured by Effort-Reward Imbalance (ERI)scale, and job satisfaction assessed by the short Chinese version of the Minnesota Satisfaction Questionnaire (MSQ) were collected in this study. A statistical analysis and hierarchical linear regression analysis were performed to explore the influencing factors and the correlations among related variables.

**Results:**

The overall mean MSQ score was 52.01 ± 13.23. Analysis of doctor satisfaction indicated that age, education, job rank, type of institution, years of working and monthly income were statistically significant (*P* < 0.05). There were negative correlation coefficients between general job satisfaction and effort/reward ratio (ERR) (*r* = -0.130, *P* < 0.001) and overcommitment (*r* = -0.615, *P* < 0.001).

**Conclusion:**

The level of job satisfaction among family doctors was considerable low. Age, education, job rank, type of institution, years of working and monthly income were influencing factors of job satisfaction. ERI and overcommitment had a negative correlation with general job satisfaction.

## Introduction

Family doctors, known as general practitioners and family physicians, are widely regarded as the “gatekeepers” of health systems and play a vital role in providing comprehensive, continuous and appropriate primary health services [1]. To date, the family doctor service policy has been implemented in over 50 countries and regions throughout the world, including the United States, the United Kingdom, Canada, Australia, etc., and has brought significant health and economic gains. China proposed family doctors and family doctor contracted services in the 1980s. In 2003, the family doctor system was carried out as a means of developing community health services [2]. In 2009, China experienced a new round of health system reform which emphasized the pressing need of establishing a sound basic medical and health service system and adopting measures to develop and strengthen community health service centres. In 2011, the Chinese government proposed the Guiding Opinions of the State Council on the Establishment of the General Practitioner System as a national indicator of family doctor services [3]. Family doctor contract services which are established by signing the contract of the service aimed to build a long-term and better medical cooperation between family doctors and the residents, so as to meet with the diversified healthcare needs and improve the residents’ health status [4, 5] have been available in Sichuan Province since 2016. However, due to the short-term development of family doctor primary care systems in China, primary care is a relatively new discipline in the medical field and a sequence of prominent defects and problems come to manifest, including an increased workload at community health service centres, an inadequate contract service rate, family doctor shortages, and the absence of supporting policies [6–8], unnecessary and tortuous paperwork, skimpy wages and salaries.

Occupational stress refers to a kind of psychological state, which refers to the response employees who experienced in response to several adverse conditions related to the working environment may have when one’s working resources, abilities and needs are not sufficient to cope with work demands and pressure [9]. It is reported that work stress prevalence rates among general working population are from 19 to 30% [10]. However, there is a high work stress prevalence among health workers at community health service centres. A survey carried out in Shanghai demonstrates that 190 (61.7%) family doctors experienced excessive occupational stress [11]. Similarly, an investigation conducted in in southwestern China showed that 78.39% primary health workers had occupational stress [12]. Occupational stress also significant impact on staff mental and psychological well-being.

The effort-reward imbalance (ERI) questionnaire is a theoretical and validated psychosocial scale widely used in exploring associations between perceived occupational stress and health state through identifying failed reciprocity between the effort spent at work and the rewards received in turn [13]. There are three dimensions: efforts, rewards and overcommitment. Work efforts refer to the demands and obligation in exposed employees. Perceived rewards represent work resources and benefits provided to the employee including money, promotion prospects, job security, respect and prestige in the workplace [14]. While Overcommitment is assessed by personal combined responses to a high effort and low reward at work [15]. The degree of occupational stress (the ERI model) can be expressed by the effort-reward ratio (ERR). If an ERR value greater than 1.0 indicated a high level of ERI. The prevalence of ERR value of > 1.0 among worker is widely prevailing, with a prevalence rate from 22.3% to 78.39% [12, 16, 17]. The ERI model is built on an imbalance between effort and reward caused a state of emotional distress from emotional and psycho-physiological stress reactions, which leads to a decrease in job satisfaction [18, 19] and an increase in burnout [20–22] and turnover [23], and subsequently results an enormous variety of adverse health outcomes [24–30].

Job satisfaction is a vital influencing indicator of one’s feeling about work, it reflects the extent to which people like their jobs [31]. To date, Job satisfaction has been assessed in diverse occupational settings, e.g., education, business firms, the military and medical care. With the prominent medical reforms and the society development, health problems and health workers’ working condition have received more and more attention. In China, a wide variety of studies have revealed the mediating effect of job satisfaction on health state and occupational commitment among nurse [32, 33], a correlation between job satisfaction and job burnout among emergency department health professionals [34, 35], the relationship between job satisfaction and turnover intention among physicians from urban state-owned medical institutions[36], the influence of effort-reward imbalance and job satisfaction on self-rated health and the way to improve job satisfaction among doctors form the public medical institutions[16, 37–39], the common reasons for dissatisfaction and job satisfaction and its related factors among psychiatry residents [40]. Few studies have focused on family doctors' job satisfaction and its related influencing factors. A survey compared the job satisfaction of community health workers (including doctors and nurses) before and after the local comprehensive medical care reform in Anhui Province and showed that the job satisfaction had a small improvement [41]. A cross-sectional survey of 448 community health workers (such as administrative staff, general practitioners, public health physicians and nurses, etc.) in Heilongjiang province suggested that there was a gap between actual and desired workplace characteristics, which had a negative influence on workers’ job satisfaction [42]. The popular researches about family doctors focused on the effect and challenge of family doctor contract services [43–48], chronic disease management to contract with family [50] and assessment of Chinese family physicians’ service competences [51]. Up to this point, these studies about job satisfaction and family doctors have been explored, however, these studies usually discussed other medical occupations or the effect of implementation family doctor policies, lacking of the overall occupational perception survey among family doctors at Community health centres. In this study, a cross-sectional survey was conducted to investigate the prevalence of job satisfaction among family doctors in Chengdu, Sichuan, China. This study not only integrated demographic characteristics, but also ERI model to examine the influencing factors of job satisfaction, the correlations between occupational stress and job satisfaction. Our study may provide a new perspective for the hospital administrators to explore effective ways of increasing family doctors’ job satisfaction.

## Methods

### Sample and setting

Chengdu is the provincial capital city of Sichuan Province and its population is about 20.9 million, which is located in southwest China. At the end of 2020, there were 2,420 family doctor teams in Chengdu, with a total of 6.9million people who signed family doctor contract services. Between March 4, 2021 and March 26, 2021, an analytical cross-sectional survey using questionnaires was conducted among family doctors through participants’ cell phones or computers to collect the data. This study was part of a larger research project, the family doctor contract services performance evaluation survey, which was sponsored by Chengdu first people’s hospital and aimed to improve community healthcare quality. Given the geographic division and economic development in Chengdu, it was divided into 23 districts and counties, including Qingyang district, high tech zone, Dayi county and Pujiang county, and so forth. Two centres were randomly selected from each geographic region, and all respondents were targeted and told about the significance and value of this survey, and reached by each centre’s administration team. As a result, a total of 46 centres and 1273 family doctors were recruited for this study. Eventually, excluding the missing records in the questionnaire and the rejection from participants, 1,105 participants completely responded anonymously (effective response rate: 86.80%). This study was approved by the ethics committee of Chengdu first people’s hospital. All subjects in this study were voluntary and expressed informed consent prior to the questionnaires.

### Instruments

#### Sociodemographic variables

Demographic and working characteristics collected included gender, age, marital status, educational levels, technical title, work contract, type of institution, years of work and monthly take-home pay. Gender was divided as ‘male’ and ‘female’. Age was categorized as ‘ ≤ 25 years old’, ‘26 ~ 35 years old’, ‘36 ~ 45 years old’, ‘46 ~ 55 years old’ and ‘ ≥ 56 years old’ five groups. Marital status was categorized as ‘single’, ‘married’, and ‘divorced or widowed’. Educational levels were divided into ‘junior college or below’, ‘bachelor’s, and ‘master’s, or above’. Technical title was classified into ‘primary title or below’, ‘middle title’, and ‘vice-senior title, or above’. Work contract was categorized into informal work contract and formal work contract. Type of institution were divided as ‘village clinic /station’, ‘township health center’, ‘community health service centres’ and ‘second-class hospital’. Years of work was divided as ‘ ≤ 5 years’, ‘6 ~ 9 years’, ‘10 ~ 15 years’, ‘16 ~ 20 years’ and ‘ > 21 years’. Monthly take-home pay was categorized as ‘1000 ~ 3000 yuan’, ‘3001 ~ 6000 yuan’, ‘6001 ~ 9000 yuan’, and ‘9001 ~ 12,000 yuan and above’.

#### Assessment of effort-reward imbalance

The 23-item effort-reward imbalance scale was applied to assess occupational stress. The questionnaire is divided into three dimensions, including effort (6 items), reward (7 items), and overcommitment (6 items). Responses to the components of “effort” and “reward” are 5-point Likert scales (1 = completely disagree, 5 = strongly agree). The component of “overcommitment” is a 4-point Likert scale (1 = strongly disagree, 4 = full agreement). The overall imbalance between effort and reward was expressed by ERR [52]. ERR was calculated as [(effort score/reward score) × 0.545], where 0.545 was the correction factor based on the number of effort items and the number of reward items (6/11). If an ERR value of > 1.0 indicated a high level of imbalance between effort and reward [16, 17].

#### Satisfaction with working conditions and work itself

We assessed job satisfaction using the short Chinese version of the Minnesota Satisfaction Questionnaire (20-MSQ short version items) [38, 53], which was developed to measure job general satisfaction along two dimensions: intrinsic satisfaction (12 items, e.g., “The chance to do different things from time to time”) and extrinsic satisfaction (6 items, e.g., “The praise I get for doing a good job”). The degree of job satisfaction was rated on a 5-point Likert scale ranging from 1 = very dissatisfied to 5 = very satisfied. The sum scores for job satisfaction were ranged from 20 to 100. Higher scores on this scale were associated with a higher level of job satisfaction.

#### Data analysis

The characteristics of variables were presented as percentages and arithmetic means and standard deviation. The scores of job satisfaction among different variables were considered as continuous variables. First, the dimensions of job satisfaction by demographic variables and ERR were verified by the Mann–Whitney U test and Kruskal–Wallis test for the non-normal continuous variables, and Student’s t-test and one-way ANOVA for the normal continuous variables. Then, correlations between job satisfaction scores and ERR and overcommitment were performed by Pearson correlation. Moreover, hierarchical linear regression analyses were performed to estimate the association between job satisfaction and demographic characteristics, ERR and overcommitment by building progressive models. Job satisfaction score was set as the dependent variable. The independent variables were entered in two steps. In step 1, the sociodemographic variables were put in the model; in step 2, ERR and overcommitment were added. All data analysis were conducted using SPSS 17.0 (SPSS China Corp., Shanghai, China) for Windows, and a two-tailed *P*-value < 0.05 was considered to be statistically significant.

## Results

### Participant characteristics

The basic characteristics of the family doctors and the mean scores of job satisfaction in the demographic categories are summarized in Table 1. In this study, a total of 1,105 participants completed the survey; 46.8% of them were male and 53.2%were female. Most respondents were less than 46 years old (39.3% were 36 ~ 45 years old and 34.5% were younger than 36 years old), and 87.8% respondents were married. In this sample, about half of the respondents had a bachelor degree or higher (50.4%). 46.5% of the participants had a middle title or higher job rank. 48.1% of them had a formal work contract. Moreover, in this survey, 11.2%, 48.5%, 34.2% and 6.1% of participants worked in village clinic / station township, health center community health service centres, second-class hospitals, respectively. Among all the respondents, 19.1% of answered respondents worked less than 6 years, 19.5% worked 6 -10 years, 19.7% worked 11 to 15 years, 10.6% worked 16 ~ 20 years, 31.1% worked ≥ 21 years. Approximately, half of respondents reported their take-home monthly pay was among 3001–6000 RMB (50.8%). In addition, the results of univariate analysis of job satisfaction in relation to the categorical variables were also shown in Table 1. 24.1% of the participants experienced occupational stress (ERR > 1) and these doctors had markedly lower scores than those with ERR ≤ 1 in job satisfaction. Consequently, significant differences were found between the groups for the following variables: age, educational level, technical title, grade of medical institutions, years of work and take-home monthly pay (*P* < 0.01).

**Table 1:** Demographic characteristics of sample and distributions of job satisfaction (*N* = 1105)

Variable	N (%)	Job Satisfaction Mean	*P*
Gender			
Males	517(46.8)	47.00 ± 18.26	0.062^a^
Females	588(53.2)	49.04 ± 17.76	
Age (years)			
≤ 25	54(4.9)	35.28 ± 17.01	0.000^b^
26 ~ 35	327(29.6)	49.98 ± 17.61	
36 ~ 45	434(39.3)	50.33 ± 17.54	
46 ~ 55	246(33.3)	42.55 ± 18.62	
≥ 56	44(4)	48.09 ± 18.01	
Marital status			
single	110(10.0)	44.67 ± 20.83	0.097
married	970(87.8)	48.51 ± 17.59	
Divorced or widowed	25(2.3)	48.09 ± 18.01	
Educational levels			0.000^b^
junior college or below	548(49.6)	42.69 ± 17.20	
bachelor	525(47.5)	52.95 ± 17.03	
master or above	32(2.9)	60.66 ± 18.01	
Technical title			0.000
primary title or below	702(63.5)	43.16 ± 16.71	
middle title	325(39.4)	55.40 ± 16.78	
vice-senior title or above	78(7.1)	57.78 ± 20.58	
Work contract			
formal work contract	531(48.1)	52.02 ± 16.73	> 0.05
informal work contract	574(51.9)	44.45 ± 18.40	
Grade of medical institutions			
village clinic / station	124(11.2)	35.31 ± 13.54	0.000
township health centres	536(48.5)	45.95 ± 17.25	
community health service centres	378(34.2)	54.61 ± 17.19	
second-class hospitals	67(6.1)	51.99 ± 19.58	
Years of work (years)			
≤ 5	211(19.1)	47.02 ± 18.40	0.000^b^
6 ~ 10	216(19.5)	46.86 ± 18.35	
11 ~ 15	217(19.7)	52.12 ± 16.70	
16 ~ 20	117(10.6)	53.16 ± 19.27	
≥ 21	344(31.1)	48.09 ± 18.01	
Monthly take-home pay (RMB)			
1000 ~ 3000	300(27.1)	28.09 ± 7.38	0.000
3001 ~ 6000	561(50.8)	50.27 ± 10.26	
6001 ~ 9000	210(19.0)	67.04 ± 15.65	
≥ 9001	34(3.1)	71.38 ± 21.48	
ERR			
≤ 1	839(75.9)	51.38 ± 17.76	0.000
> 1	266(24.1)	37.69 ± 14.54	

The difference was examined by Student’s t test and one-way ANOVA. ^a^Mann-Whitney U test and ^b^Kruskal-Wallis test were applied for the non-normal continuous variables

### Correlation between occupational stress and job satisfaction

The means, standard deviations (SD), and results of the Pearson correlation analyses are detailed in Table 2. Intrinsic job satisfaction showed negative correlation with ERR (*P* < 0.001), Intrinsic job satisfaction had a comparatively higher correlation coefficient with overcommitment (*r* = -0.615, *P* < 0.001). A negative significant correlation was observed between extrinsic job satisfaction and overcommitment (*r* = -0.433, *P* < 0.001). When the correlation between general job satisfaction and occupational stress was examined, there were negative correlation coefficients between general job satisfaction and ERR (*r* = -0.130, p < 0.001) and overcommitment (*r* = -0.615, *P* < 0.001).

**Table 2:** Correlation between occupational stress and job satisfaction

	Mean	SD	1	2	3	4	5
1ERR	0.91	0.288	1				
2Overcommitment	17.02	2.54	0.113**	1			
3Intrinsic job satisfaction	32.08	9.01	-0.163**	-0.615**	1		
4Extrinsic job satisfaction	15.72	4.28	-0.030	-0.433**	0.563**	1	
5General job satisfaction	52.01	13.23	-0.130**	-0.615**	0.954**	0.782**	1

^**^ *P* < 0.001, effort/reward ratio (ERR)

### Factors associated with job satisfaction

The data were further employed for hierarchical regression analyses using job satisfaction as the dependent variable, where the sociodemographic characteristics and occupational stress were taken as independent variables. The major factors associated with job satisfaction are presented in Table 3. In the first step, sociodemographic factors accounted for 39.0% of the variance in intrinsic job satisfaction, 72.9% of the variance in extrinsic job satisfaction, 60.9% of the variance in overall job satisfaction. In the second step, the dimensions of occupational stress explained 52.4% of the variance in intrinsic job satisfaction, 68.3% of the variance in overall job satisfaction. However, gender, age, marital status, work contract, and grade of medical institutions had no significant association with all dimensions of job satisfaction in the hierarchical linear regression analyses.

**Table 3:** Hierarchical linear regression analyses for exploring associated factors for job satisfaction

Variables	Intrinsic Job Satisfaction		*Extrinsic Job Satisfaction*		General Job Satisfaction	
	Step 1 (β)	Step 2 (β)	Step 1 (β)	Step 2 (β)	Step 1 (β)	Step 2 (β)
Gender	0.029	0.001	-0.031	-0.032	0.015	-0.008
Age	0.034	0.034	-0.026	-0.026	0.003	0.004
Marital status 1	-0.037	-0.027	-0.019	-0.018	-0.028	-0.017
Marital status 2	0.002	0.014	-0.001	-0.001	0.000	0.007
Educational levels 1	-0.014	-0.011	0.038*	0.038*	0.006	0.007
Educational levels 2	0.038	0.002	0.002	0.001	0.029	0.002
Technical title 1	0.028	0.015	-0.019	-0.020	0.013	0.002
Technical title 2	0.089**	0.063**	-0.003	-0.004	0.067**	0.041*
Work contract	-0.030	-0.060	-0.002	-0.001	-0.022	-0.044
Grade of medical institutions 1	-0.005	-0.019	0.026	0.035	-0.003	-0.014
Grade of medical institutions 2	-0.027	0.001	0.009	0.011	-0.015	0.007
Grade of medical institutions 3	0.029	0.019	0.081	0.009	0.026	0.023
Years of work 1	-0.030	-0.029	-0.074**	-0.074**	-0.046*	-0.046**
Years of work 2	-0.052**	-1.497*	-0.041*	-0.041*	-0.054**	-0.049**
Years of work 3	0.005	-0.016	0.009	0.009	0.002	-0.014
Years of work 4	0.016	0.028	0.020	0.021	0.004	0.014
Take-home monthly pay1	0.799**	0.539**	1.395**	1.380**	1.118**	0.844**
Take-home monthly pay 2	0.363**	0.242**	0.970**	0.956**	0.636**	0.454**
Take-home monthly pay3	0.002	0.019	0.357**	0.350**	0.121**	0.041
ERR		-0.077**		0.022		-0.300**
Overcommitment		-0.400**		-0.017		-0.046**
Adjusted R^2^	0.390	0.521	0.729	0.729	0.609	0.680
△R^2^	0.392	0.524	0.730	0.731	0.611	0.683

*ERR* Effort–reward ratio. Marital status1: married vs. Single; Marital status 2:married vs. divorced/widowed; Educational levels 1: bachelor vs. junior college or below; Educational levels 2: bachelor vs. master and above; Technical title 1:middle title vs. primary title or below; Technical title 2: middle title vs. vice-senior title or above; Grade of medical institutions 1: community health service centres vs. village clinic/station; Grade of medical institutions 2: community health service centres vs. township health centres; Grade of medical institutions 3: community health service centres vs. second-class hospitals; Years of work 1: 11 ~ 15 years vs. ≤ 5 years; Years of work 2:11 ~ 15 years vs.6 ~ 10 years; Years of work 3: 11 ~ 15 years vs. 16 ~ 20 years; Years of work 4:11 ~ 15 years vs. ≥ 21 year; Take-home monthly pay1: 6001 ~ 9000 yuan RMB vs.1000 ~ 3000 yuan RMB; Take-home monthly pay 2: 6001 ~ 9000 yuan RMB vs. 3001 ~ 6000 yuan RMB; Take-home monthly pay 3: 6001 ~ 9000 yuan RMB vs ≥ 9001 yuan RMB. * *P* < 0.05, ** *P* < 0.01

## Discussion

In the present study, 1,105 participants were sampled from 46 health centres in 23 districts and towns throughout Chengdu and completed the survey, and they experienced a low level of job satisfaction (the overall mean MSQ score was 52.01 ± 13.23), which was lower than that of Chinese university teachers (69.71), Chinese township cadres (71.21), community health workers from two cities in northern China (68.2) and Chinese specialists(65.86)[17, 18, 38, 39, 53]. A possible explanation for the discrepancy is China’s national conditions and its geographical and economic diversity. Therefore, there is still a large room for improving job satisfaction among family doctors.

In this study, age, education, job rank, institution type, years of working and monthly income are demographic factors of family doctors’ job satisfaction, which are congruous with previous studies [39, 54–57]. With respect to age, a study conducted among university teachers revealed a U-shaped relationship between age and job satisfaction [58], but our study did not report such kind of relationship. Job satisfaction is much greater in the 36 to 45 years old group than in the other age groups. The explanation might be that this group had a high education level and climbed rapidly up the professional ladder, enabling them to be content with their job accomplishment. Family doctors with a master’s degree and above scored higher than those with a bachelor’s degree and below. Family doctors with a high education level can get access to publishing academic papers and undertaking research projects, which are the basic requirements for applying for a promotion in China. The current study showed that a worker’s professional rank influenced the level of job satisfaction, which was consistent with other studies [59, 60]. In addition, participants who worked in community health service centres were more likely to report a higher score of job satisfaction than those who worked in other types of health institutions. A possible explanation was that community health centres located in economically developed areas had a greater opportunity to improve healthcare access, basic health infrastructure, and high-quality health services than others. As for working years, some studies found that they affected staff satisfaction [39, 61, 62], but others presented the opposite results [63]. In the present study, workers with 16–20 working years were more satisfied with their job. The effect of working years was also reflected in the regression model. The reason for this phenomenon could be that staff with a longer duration of working experience were equipped with adequate medical skills and were responsible for more work, resulting in higher salary and social status. Moreover, we reported that sufficient monthly income was a strong predictor of job satisfaction, which was consistent with these previous findings [61, 64]. But there were other points of view on monetary factors. Studies abroad indicated that financial and non-financial factors affected job motivation and satisfaction among rural health workers [65] and that non-monetary factors had a greater impact on professional and performance satisfaction than income among health workers [66, 67]. Hence, income escalation combined with non-financial incentives could improve multidimensional satisfaction among family doctors.

This study demonstrated that effort-reward imbalance and overcommitment had a detrimental effect on general job satisfaction in correlation analysis. In addition, we found that 24.1% of the participants experienced occupational stress (ERR > 1). Moreover, overcommitment (rather than effort-reward imbalance) was a strong determinant of family doctors’ job satisfaction. According to the hierarchical regression results, based on the absolute value of β, effort-reward imbalance and overcommitment were likewise negatively correlated with job satisfaction. With the initiation of China’s new medical reform and health care system, family doctors assume the role as gatekeepers of the public’s health. Community health service policies, including family doctor contract services and hierarchical diagnosis and treatment, have reaped benefits in terms of reducing the difficulty of accessing medical services and its high costs and solving other health issues. However, the family doctor contract services were launched and developed later in our region than in other areas in China. Family doctors exerted greater effort toward organizational overall goals, suggesting that they might overestimate their abilities and devote more effort to tasks that were beyond their capabilities, leading to failed reciprocity between efforts and rewards. Moreover, due to the huge population and rapid ageing of the population, an endless number of rules and regulations, a lack of promotion and learning opportunities, and poor wages and salaries, community doctors are working in a high-stress environment and experiencing decreasing job satisfaction [49, 68, 69].Research suggested that employees with the effort-reward imbalance and high overcommitment were at a higher risk of stress-related mental and physical distress illnesses [63, 70] which resulted in decreasing job satisfaction. In addition, lowering the job satisfaction at workplace leads to a low sense of belonging and enthusiasm for work, as well as to a decreasing organization’s retention rate [71, 72]. On the contrary, a high level of job satisfaction has been shown to improve psychological and mental well-being [73]. Therefore, the requirements for basic health services at community health centres in China are increasing as the economy grows and the population ages. Notably, in the context of preventing and controlling the COVID-19 epidemic, family doctors must assume increasing responsibility and multitasking works, which caused the primary healthcare workers to feel stressed and depressed [74]. Research reported that challenges in carrying out the responsibility and multitasking work had a crippling effect on job satisfaction [41, 61].

Our study showed that family doctors suffered great work pressure, put more effort into completing work with lower rewards, and complained about low job satisfaction. We value the importance of family doctors considered as the backbone of basic healthcare in China. Therefore, it is imperative to take multiple measures to change the current situation. It is necessary to establish an effective hierarchical diagnosis and treatment system, to incent the specialists from general hospitals to help family doctors, to provide more learning and training opportunities, so as to improve their clinical competence. In addition, policy-makers need pay more attention to family doctors’ environment and conditions to formulate efficient policy and to meet their rational demands. Furthermore, healthcare managers motivate current staff by increasing income level, ameliorating promotion requirements, and enabling worker to be masters of their own decisions to avoid turnover intention and ensure the stability of the primary health workforce.

Furthermore, our study may provide a new perspective for the health administrators so that they could promote family doctors’ job satisfaction by developing strategic changes. To mitigate the dissatisfaction of family doctors at primary healthcare centres, it is necessary to establish a better incentive system and modify working conditions. This can be done by increasing income level, ameliorating the work burden, providing more learning and training opportunities, improving education level and professional title, avoiding turnover intention and ensuring the stability of the primary health workforce.

On the other hand, several limitations of this study also should be considered. First, due to the characteristics of a cross-sectional design in this study, it was not able to draw causal conclusions. To deepen the understanding of job satisfaction in this study, a qualitative approach or a longitudinal study is needed. Second, some other variables that have not been considered in this study might also have an impact on job satisfaction. Therefore, further research is needed to verify the relationship between these variables and job satisfaction. Third, although the participants were form every geographical area in Chengdu, owing to some constraints of time and convenience, the study results may not draw some extrapolated conclusion to generalize the findings to other cultures and geographic regions in China.

This study revealed that the level of job satisfaction among family doctors was considerable low. Age, education, job rank, type of institution, years of working and monthly income were influencing factors of job satisfaction. There was negative significant association between effort-reward imbalance, overcommitment and general job satisfaction. The results have implications for interventions to improve the job satisfaction of family doctors. A balance between efforts and rewards, a better incentive system and modify working conditions should put forward to increase family doctors’ job satisfaction.

## Data Availability

The data and materials in this study are available from the corresponding author on reasonable request.
